# Yellow Fever Virus in *Haemagogus leucocelaenus* and *Aedes serratus* Mosquitoes, Southern Brazil, 2008

**DOI:** 10.3201/eid1612.100608

**Published:** 2010-12

**Authors:** Jáder da C. Cardoso, Marco A.B. de Almeida, Edmilson dos Santos, Daltro F. da Fonseca, Maria A.M. Sallum, Carlos A. Noll, Hamilton A. de O. Monteiro, Ana C.R. Cruz, Valéria L. Carvalho, Eliana V. Pinto, Francisco C. Castro, Joaquim P. Nunes Neto, Maria N.O. Segura, Pedro F.C. Vasconcelos

**Affiliations:** Author affiliations: Secretaria da Saúde do Estado do Rio Grande do Sul, Porto Alegre, Brazil (J. da C. Cardoso, M.A.B. de Almeida, E. dos Santos, D.F. da Fonseca, C.A. Noll);; Universidade de São Paulo, São Paulo, Brazil (J. da C. Cardoso, M.A.M. Sallum);; Instituto Evandro Chagas, Ananindeua, Brazil (H.A. de O. Monteiro, A.C.R. Cruz, V.L. Carvalho, E.V. Pinto, F.C. Castro, J.P. Nunes Neto, M.N.O. Seguara, P.F.C. Vasconcelos);; Universidade do Estado do Pará, Belém, Brazil (P.F.C. Vasconcelos)

**Keywords:** TOC summary: *Hg. leucocelaenus* is the main vector, *Ae. serratus* may be a secondary vector, Yellow fever, Haemagogus leucocelaenus, Aedes serratus, arbovirus surveillance, entomologic surveillance, mosquitoes, Brazil, research

## Abstract

Yellow fever virus (YFV) was isolated from *Haemagogus leucocelaenus* mosquitoes during an epizootic in 2001 in the Rio Grande do Sul State in southern Brazil. In October 2008, a yellow fever outbreak was reported there, with nonhuman primate deaths and human cases. This latter outbreak led to intensification of surveillance measures for early detection of YFV and support for vaccination programs. We report entomologic surveillance in 2 municipalities that recorded nonhuman primate deaths. Mosquitoes were collected at ground level, identified, and processed for virus isolation and molecular analyses. Eight YFV strains were isolated (7 from pools of *Hg. leucocelaenus* mosquitoes and another from *Aedes serratus* mosquitoes); 6 were sequenced, and they grouped in the YFV South American genotype I. The results confirmed the role of *Hg. leucocelaenus* mosquitoes as the main YFV vector in southern Brazil and suggest that *Ae. serratus* mosquitoes may have a potential role as a secondary vector.

Yellow fever is an acute, often fulminant, disease caused by *Yellow fever virus* (YFV), the prototype member of the family *Flaviviridae*, genus *Flavivirus*. YFV is endemic to tropical regions of Africa and South America (*1**,**2*). The virus is transmitted through the bite of mosquitoes belonging to the family *Culicidae* to vertebrate hosts, especially nonhuman primates and humans.

In South America, the urban cycle involves the mosquito *Aedes aegypti* and humans, whereas in the jungle cycle, the virus is transmitted to nonhuman primates by mosquitoes in the genera *Haemagogus* and *Sabethes*, especially *Hg. janthinomys*, *Hg. albomaculatus*, *Hg. leucocelaenus*, *Sa. chloropterus*, *Sa. glaucodaemon*, *Sa. soperi*, and *Sa. cyaneus* (*2**,**3*).

Currently in Brazil, 2 yellow fever–endemic areas have been described. The area where vaccination is recommended or risk for yellow fever is recognized includes the northern and central regions, as well as Maranhão State and the eastern part of Bahia, Minas Gerais, São Paulo, Paraná, Santa Catarina, and Rio Grande do Sul states. The area where vaccination has not been recommended includes the coastal region between Piauí and Rio Grande do Sul states (*4*). During 1989–2008, a total of 546 human cases of yellow fever and 241 deaths were recorded in Brazil (*5*).

In Rio Grande do Sul, the southernmost state in Brazil, the last cases of sylvatic yellow fever were recorded in the 1960s (*6*). After 40 years without detectable activity, the virus was isolated from mosquitoes (*Hg. leucocelaenus)* collected in 2001, during an epizootic involving free-living nonhuman primates of the species *Alouatta caraya* (black howler monkey) in the northwestern region of the state (*7*). These YFV hosts spend most of their time in the trees and only go down to the ground to feed during the day; they are extremly sensitive to YFV and die of the disease after they are infected naturally or experimentally even in lower doses.

The confirmation of YFV in nonhuman primates and in mosquitoes led to vaccination campaigns in 44 municipalities to prevent human cases and the initiation of a program of environmental surveillance for yellow fever and other arboviruses authorized by Brazil’s state Ministry of Health. The goal of this program was to detect the early presence of YFV in mosquitoes and nonhuman primates (through the detection of specific antibodies). The monitoring program was improved and, in 2002, a new epizootic was recorded with virus circulation in the central region of the state, including 9 additional municipalities in the vaccination area. Subsequently, no YFV activity was recorded for 6 years.

In October 2008, the state health secretary reported an increase in the number of deaths of black howler monkeys in the northwestern region, and intensified surveillance began immediately, before the deaths from yellow fever were even confirmed (*8*). This study describes the results obtained in 2008 during entomologic surveillance in areas with records of yellow fever epizootics in 2 municipalities of the northwest region of Rio Grande do Sul State.

## Materials and Methods

### Study Area

**Figure 1:**
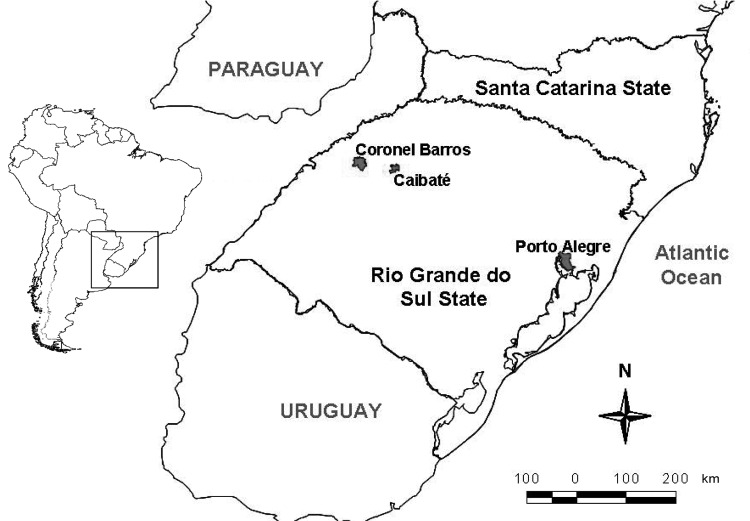
Municipalities in Rio Grande do Sul State, southern Brazil, where mosquito specimens were collected during November 2008.

Entomologic activities were carried out in rural areas in Caibaté (54°38′W, 28°17′S) and Coronel Barros (54°03′W, 28°22′S) municipalities in the Ijuí River basin. These 2 municipalities are located 487 km and 417 km, respectively, from Porto Alegre, the Rio Grande do Sul State capital, and are ≈70 km from each other (Figure 1). Caibaté has 5,080 inhabitants, and Coronel Barros has 2,241 inhabitants, according to a 2007 census (*9*). The landscape is dominated by plantations and extensive ranching, which demonstrates the extensive human influence on the environment (*10*).

Remnant tree formations are distributed in gallery forests or in isolated forest fragments in the cultivated areas and in pasture grasslands generally associated with small rivers and creeks that offer shaded areas and water for cattle. Forests in the region are classified as deciduous stationary forest. This category is defined by the low temperatures observed during the winter, which cause the trees in the upper strata to lose their leaves because of physiologic drought caused by the cold weather (*11*).

The climate is humid subtropical, with 2 distinct seasons during the year. During the summer, the mean temperature is >20°C; during the winter, the mean temperature drops <15°C. In relation to rainfall, there are no periods with water deficit (*12*)*.*

### Field Procedures

Samples were obtained during November 19–27, 2008, in residual forests where nonhuman primate deaths had been recorded in Caibaté and Coronel Barros municipalities. Mosquitoes were captured at ground level by using entomologic net and bottle-type manual vacuums, at different times, from 8:30 am to 4:00 pm. After sampling, the insects were frozen, transferred to cryogenic tubes, and placed in liquid nitrogen containers for transportation to the laboratory where they were stored in a freezer at –70°C until processing. A total of 358 mosquitoes were captured in Coronel Barros (n = 179; 16 pools) and Caibaté (n = 179; 20 pools) for virus isolation attempts (Table 1).

**Table 1:** Mosquitoes used for yellow fever virus isolation in municipalities of Coronel Barros and Caibaté, Rio Grande do Sul State, Brazil, November 2008

Taxonomic category	No. mosquitoes (no. batches)						Total
	Coronel Barros				Caibaté		
	Rincão dos Pampas	Rincão Canta Galo	Linha da Pedreira		Linha Caaró	Capão do Herval	
*Aedes scapularis*					1		1
*Ae.serratus*	8	3	4		32	6	53 (5)
*Anopheles mediopunctatus*					1		1
*Chagasia* sp.	2					1	3 (2)
*Coquillettidia venezuelensis*					1		1
*Haemagogus leucocelaenus*	56 (2)	83 (3)			36 (2)	14	189 (8)
*Johnbelkinia* sp.					1		1
*Psorophora albigenu*						1	1
*Ps. albipes*					3	38 (2)	41 (3)
*Ps. ferox*	1	1	3		11	12	28 (5)
*Psorophora* sp.	6	7					13 (2)
*Sabethes albiprivus*					5		5
*Sa. intermedius*	2				1		3 (2)
*Wyeomyia* sp.		3			13	2	18 (3)
Total	75 (7)	97 (7)	7 (2)		105 (12)	74 (8)	358 (36)

### Taxonomic Identification and Viral Isolation

#### Cell Culture

Batches of mosquitoes were identified to species or genus level, separated into pools of 30 individuals (maximum), and macerated in 1.0 mL of phosphate-buffered saline solution with bovine albumin, containing penicillin (100 IU/mL) and streptomycin (100 µg/mL). The pools were then centrifuged at 3,000 rpm at 4°C for 10 min, filtered, and added to cultures of Vero cells. The cells were observed for 14 days; after this period, cultures were harvested and supernatants used in indirect immunofluorescent assays with polyclonal antibodies against flaviviruses (Bussuquara, Cacipacore, dengue, yellow fever, Ilhéus, Naranjal-like, Rocio, and Saint Louis encephalitis viruses) and alphaviruses (Aura, eastern equine encephalitis, Mayaro, Mucambo, Pixuna, Una, western equine encephalitis, and Trocara viruses). Mosquito batches positive for flaviviruses were retested by using indirect immunofluorescent assays with monoclonal antibodies from the identification strain isolated (*7**,**13*).

### Newborn Mice

Newborn Swiss albino mice were inoculated by the intracerebral route with 0.02 mL of the same suspension used to infect cell cultures. Mice that demonstrated signs of disease were removed and tested by a complement-fixation technique to confirm virus isolation as described (*14**,**15*).

### Minimum Infection Rate

The minimum infection rate in the mosquito species from which YFV has been isolated was calculated as follows. The total number of groups of positive species of mosquito was divided by the total number of processed mosquitoes of that species × 100 (*16*).

### RNA Extraction and Reverse Transcription–PCR

To extract viral RNA, the reagent Trizol LS protocol (Invitrogen, San Diego, CA, USA) was used, following the manufacturer’s instructions. The cDNA was obtained directly from viral RNA by reverse transcription in vitro for the envelope (E) region. Five microliters of RNA was used and added to 1 μL (0.2 μmol/L) of the reverse oligonucleotide primer FA2554 (5′-GTATGAGTACTTGTTCAGCCAGTC-3′). This mixture was incubated for denaturation of the RNA molecule at 94°C for 2 min and at room temperature for 5 min. Next, , 14 μL of the RT solution, containing 1× buffer, 1 mmol/L of each, 10 mmol/L dithiothreitol, 40 U RNase inhibitor (RNaseOUT; Invitrogen), and 1.5 U reverse transcriptase (Superscript-II, Reverse Transcriptase; Invitrogen) were added. cDNA synthesis was carried out at 45°C for 1 h. The solution was then heated at 94°C for 10 min and incubated at 4°C until the addition of the PCR mixture for cDNA amplification.

To the final volume of 20 μL of reverse transcription were added 30 μL of the PCR mixture, which had 1× buffer, 2 mmol/L MgCl_2_, 2.5 U DNA polymerase (Platinum Taq DNA Polymerase), 0.2 µmol/L of oligonucleotides forward (FA1223) (5′-GAAGAGAACGAAGGGACAATGC-3′) and reverse (FA2554) primers to sequencing of the YFV E region. The program used for amplification consisted of a previous denaturation at 94°C for 2 min, followed by 35 cycles, each one composed of denaturation steps at 94°C for 30 s, hybridization at 55°C for 30 s, and extension at 72°C for 2.5 min, followed by a final extension cycle at 72°C for 5 min.

### Nucleotide Sequencing

For nucleotide sequencing, the amplicons were purified by using the QIAquick Gel Extraction Kit (QIAGEN, Valencia, CA, USA), which uses silicon retention columns, following the protocol described by the manufacturer. The purified cDNA was sequenced by using the ABI PRISM Dye Terminator kit version 3.1 (Applied Biosystems, Foster City, CA, USA), which uses the chain termination methods with dideoxyribonucleotides labeled with different fluorophores for each nucleotide on the 3′ end (*17*).

In the mixture for each sequencing reaction, we used 8 μL of Terminator ready reaction mixture, 2 μL (100 ng) of the PCR product, 3.5 pmol/L of the oligonucleotides FA1223 and FA2554, and water to give a final volume of 10 μL. The sequencing reaction product was precipitated by using an ethanol and isopropanol protocol and subjected to electrophoresis in an automated sequencer ABI PRISM 3130 (Applied Biosystems).

### Phylogenetic Analyses and Sequences Obtained

To align and analyze the homology of the sequences obtained from the E gene, we used the computer programs SEQMAN II, EDITSEQ, and MEGALIGN included in the Lasergene package version 4.05 (DNASTAR Inc., Madison, WI, USA) were used. To construct the dendrograms and phylogenetic trees of envelope region, the Molecular Evolutionary Genetics Analysis program, version 4.1 (*18*) was used, applying the neighbor-joining method. The nucleotide distance was calculated by using the Kimura 2-parameter method and bootstrap analyses with 1,000 pseudoreplicates (*19*). To enable better rooting of the phylogenetic tree, we added the strain ASIBI (YFV prototype) and a strain of YFV from Uganda as outgroups.

## Results

A total of 358 mosquitoes belonging to 14 taxonomic categories were collected. The most abundant species were *Hg. leucocelaenus* (52.8%) and *Aedes serratus* (14.8%).

From the 189 specimens of *Hg. leucocelaenus* captured, 6 (91.5%) of 7 pools were positive for YFV. YFV was not detected in 1 pool which contained 16 mosquitoes from Linha Caaró County. The calculated minimum infection rate was 1.88% for *Ae. serratus* and 3.70% for *Hg*. *leucocelaenus*.

YFV was isolated from 7 pools of *Hg. leucocelaenus* mosquitoes collected in Coronel Barros and Caibaté. Moreover, an additional YFV isolate was obtained from a pool of 3 *Ae. serratus* mosquitoes sampled in Coronel Barros (Table 2). All YFV-positive pools showed positive results with the 3 techniques used, except strain BeAr 754962, which was only positive by cell culture and by reverse transcription–PCR.

**Table 2:** Mosquitoes positive for yellow fever virus, by lot number, collected in Coronel Barros and Caibaté municipalities, Rio Grande do Sul State, Brazil, November 2008

Date	Time	Municipality	Locality	Lot	Species	No.
25	10:00 AM–2:00 PM	Coronel Barros	Rincão Canta Galo	754954	*Haemagogus leucocelaenus*	30
25	10:00 AM–2:00 PM	Coronel Barros	Rincão Canta Galo	754955	*Hg. leucocelaenus*	30
26	10:05 AM–2:15 PM	Coronel Barros	Rincão Canta Galo	754956	*Hg. leucocelaenus*	23
25	10:00 AM–2:00 PM	Coronel Barros	Rincão Canta Galo	754957	*Aedes serratus*	03
19	8:30 AM–11:30 AM	Coronel Barros	Rincão dos Pampas	754962	*Hg. leucocelaenus*	29
27	9:40 AM–1:40 PM	Coronel Barros	Rincão dos Pampas	754963	*Hg. leucocelaenus*	27
26	9:30 AM–12:00 PM	Caibaté	Capão Herval	754984	*Hg. leucocelaenus*	14
25	9:00 AM–4:00 PM	Caibaté	Linha Caaró	754993	*Hg. leucocelaenus*	20

**Figure 2:**
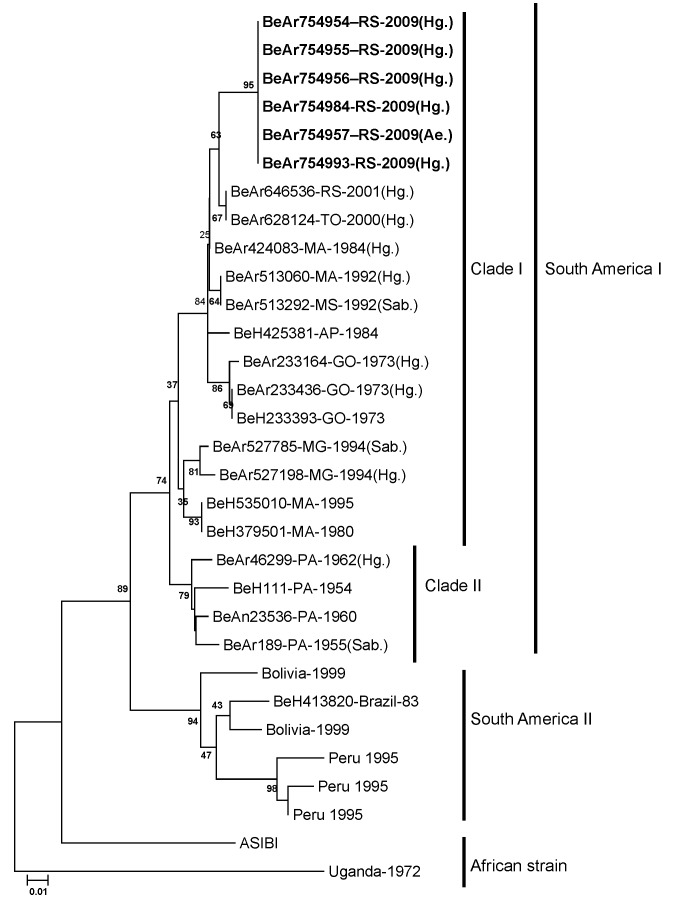
Phylogenetic analysis of partial (1,205 nt) structural region of the envelope gene of 6 yellow fever virus (YVF) isolates (**boldface**) sequenced from samples recovered from hematophagous arthopods collected in Rio Grande do Sul State, southern Brazil, November 2008. Comparison is shown with sequences of 17 genotype I YFV strains from Brazil and with sequences of 6 reference strains of genotype II from South America (Peru, Bolivia, and Brazil) obtained from GenBank. The analysis was performed by the neighbor-joining method; the nucleotide distance was calculated by the Kimura 2-parameter method. Bootstrap values were calculated after 1,000 replicates and are listed only in the main branches. The sequences of YFV strain Asibi and Uganda were used as outgroups. Scale bar indicates a divergence of 10%.

A phylogenetic analysis with 6 YFV isolates recovered from hematophagous arthopods collected in Rio Grande do Sul (5 from *Hg. leucocelaenus* and 1 from *Ae. serratus*) was carried out after 1,205 nt of E gene were sequenced and compared with sequences of other YFV strains from South America, including a nucleotide sequence of an isolate of *Hg. leucocelaenus* collected in 2001 in Rio Grande do Sul State. The 2008 YFV strain that circulated in the state during the yellow fever outbreak belongs to the South American I genetic lineage. Although it was the same genotype as YFV from *Hg. leucocelaenus/*2001, it was genetically distinct (Figure 2).

## Discussion

Yellow fever still represents an infectious disease that causes a high proportion of illness and death. Forty years since the last human yellow fever case in Rio Grande do Sul state, an epizootic was reported in nonhuman primates; the virus was isolated from mosquitoes in southern Brazil in 2001 with no record of infection in humans (*7*). Surprisingly, specimens of *Hg. janthinomys*, considered the main sylvan vector of the YFV in the Americas, were not collected in this region, and *Hg. leucocelaenus* was the most abundant species captured.

*Hg. leucocelaenus* is a broadly distributed species in South America, although its presence has not yet been documented on the west side of the Andes or in southern Chile and Argentina. This species is found in forests and has diurnal and acrodendrophilic habits, although the mosquito can take blood from hosts at ground level (*20*). In Brazil, this species is most abundant and common in forests in the southern region (*21*).

In Rio Grande do Sul State, *Hg. leucocelaenus* was first documented in the municipality of Taquara in 1932 (*22*). In 1939, it was detected in Santa Rosa, Santo Augusto, and São Luiz Gonzaga municipalities in the northwestern region of the state (*21*).

YFV was isolated from *Hg. leucocelaenus* mosquitoes in 1938 from 16 specimens collected in Rio de Janeiro State (*23*) and in 1944 from a pool with 6 specimens from a forest close to Villavicencio, Colombia (*21*). Sixty years later, during an epizootic in rural areas in the northwestern region of Rio Grande do Sul State, 2 strains of YFV were isolated from 21 specimens of these mosquitoes in a pool with 6 insects captured in the canopy and another pool of 15 *Hg. leucocelaenus* mosquitoes captured at ground level in Santo Antonio das Missões municipality (*7*). At that time, researchers suggested that *Hg. leucocelaenus* mosquitoes could be a secondary vector of YFV, in addition to having a major role in the epidemiology of this arbovirus in the Southern Cone region of South America.

A biologic vector can be classified as a main or primary vector or as an auxiliary or secondary vector, according to the transmission area it is associated with. According to Forattini (*20*), auxiliary vectors contribute to the action of the main vectors when they coexist with, or can take the role of, the main vector, but at a local or regional level. In contrast, the main vectors fulfill this role in broad biogeographic regions.

Until now, *Hg*. *leucocelaenus* has been the only species in the genus with a confirmed presence in Rio Grande do Sul (*24*). Thus, our data support the role of *Hg. leucocelaenus* mosquitoes as the primary vector of the sylvan yellow fever in the state. However, this finding could be broadened to include other states in the southern Brazil.

In São Paulo State, entomologic investigations (*25*) in several municipalities verified that *Hg. leucocelaenus* was one of the most abundant and most frequently captured species in all studied regions during an outbreak of sylvan yellow fever in 2001, but YFV was only isolated from *Hg*. *janthinomys* mosquitoes. The authors of that study demonstrated the capacity of *Hg. leucocelaenus* to adapt to secondary and degraded environments and, although they collected mosquitoes of other species (*Hg*. *janthynomis*/*capricornii* and *Hg*. *spegazzinii*), they highlighted the possibility that *Hg*. *leucocelaenus* mosquitoes might also be involved in the maintenance cycle of YFV in the area (*25*).

All mosquitoes obtained in Caibaté and Coronel Barros municipalities were collected close to the ground in remnants of deciduous forests, surrounded by soy plantations. Because the continuous canopy of these forests does not exceed 20 m (*12*), and because of the low flight range of *Hg. leucocelaenus* mosquitoes associated with the active search for blood demonstrated by females (*20*), sampling mosquitoes at ground level likely enables collection of an a sizeable number of individuals for taxonomic identification and viral isolation. Thus, canopy platforms are not needed, as they are in other regions of the country, to collect *Hg. janthinomys* and other species. In 2001, when YFV was isolated in 2 pools with 23 mosquitoes, 22 specimens of *Hg. leucocelaenus* were collected at ground level (*7*).

At the Evandro Chagas Institute in northern Brazil (eastern Amazon region), ≈98% of all YFV isolates have been obtained from mosquitoes in the genera *Haemagogus* and *Sabethes*. Only occasionally were species from other genera such as *Ae. fulvus*, *Ae. scapularis*, and *Psorophora albipes* found to be infected, but with only 1 isolate (*2*); thus, these genera lack importance in the maintenance of YFV in nature.

At the Rincão Canta Galo locality, in Coronel Barros municipality, 3 specimens of *Ae. serratus* were collected from which YFV was isolated (strain BEAR 754957). These mosquitoes inhabit temporary puddles on the ground, are abundant in forest environments, and their feeding activity is diurnal with peaks at dusk. Although the females’ choice of blood meals is eclectic, they prefer to take blood from large mammals (*20**,**26*).

Records indicating that mosquito species have been naturally infected with other arboviruses suggest that this species can successfully maintain and transmit other pathogens. *Ae. serratus* mosquitoes have been found to be naturally infected with Oropouche virus in the Amazon Region (*27*); with Aura virus in Pará, Brazil, and in Misiones Province, Argentina (*28**,**29*); and with Trocara virus in the Amazon regions of Brazil (Pará State) and Peru (*30**,**31*). Moreover, *Ae. serratus* mosquitoes are considered a secondary vector of Ilheus virus (*27*).

In relation to the molecular study, the distribution of YFV isolates in the present study was monophyletic; they formed a subgroup inside clade I of the South American genotype I. Notably, however, 2 clades (I and II) grouped randomly with strains from different states in Brazil. From these results, we can hypothesize that clades have been formed on the basis of the date of virus circulation and not on the basis of their geographic distribution. Isolates from southern Brazil show an elevated genetic divergence when compared with strains in clade II, designated “Old Pará” by Vasconcelos et al. (*33*). These results were previously found in the analyses of the junction region N terminal of the nonstructural protein 5/nontranslated 3′ region of 79 samples of YFV from Brazil (*33*).

Thus, the YFV isolates from our study are genetically distinct from other YFV isolates from humans, mosquitoes, and nonhuman primates obtained in other states in Brazil during the 1960s, 1970s, 1980s, and 1990s. An interesting result was that the isolates of the present study showed a homology of 83% with an isolate of YFV from a *Haemagogus* mosquito collected in Tocantins in 2000 (*32*) and with a YFV strain recovered in Rio Grande do Sul in 2001 (*7*).

In summary, this study confirmed the key role of *Hg. leucocelaenus* mosquitoes as a vector of yellow fever in Rio Grande do Sul State. It also demonstrated the natural infection of *Ae. serratus*, which suggests that the latter species might serve as a potential secondary vector of YFV in southern Brazil, and identified that the genotype I South American, clade I was responsible for the yellow fever outbreak and epizootic in Rio Grande do Sul State in 2008. Finally, comparative studies with strains of YFV from primates and humans from the same geographic region should be conducted, using molecular clock analysis to understand the transmission dynamics of the virus.
